# Perspectives on Cannabis-Based Therapy of Multiple Sclerosis: A Mini-Review

**DOI:** 10.3389/fncel.2020.00034

**Published:** 2020-02-19

**Authors:** Miriam Mecha, Francisco J. Carrillo-Salinas, Ana Feliú, Leyre Mestre, Carmen Guaza

**Affiliations:** ^1^Departamento de Neurobiología Funcional y de Sistemas, Grupo de Neuroinmunología, Instituto Cajal, CSIC, Madrid, Spain; ^2^Department of Immunology, Tufts University School of Medicine, Boston, MA, United States

**Keywords:** cannabinoids, endocannabinoids, immunomodulation, neuroprotection, oligodendrocyte, astrocyte, microglia, multiple sclerosis

## Abstract

The consistency, efficacy, and safety of cannabis-based medicines have been demonstrated in humans, leading to the approval of the first cannabis-based therapy to alleviate spasticity and pain associated with multiple sclerosis (MS). Indeed, the evidence supporting the therapeutic potential of cannabinoids for the management of pathological events related to this disease is ever increasing. Different mechanisms of action have been proposed for cannabis-based treatments in mouse models of demyelination, such as Experimental Autoimmune Encephalomyelitis (EAE) and Theiler’s Murine Encephalomyelitis Virus-Induced Demyelinating Disease (TMEV-IDD). Cells in the immune and nervous system express the machinery to synthesize and degrade endocannabinoids, as well as their CB1 and CB2 receptors, each mediating different intracellular pathways upon activation. Hence, the effects of cannabinoids on cells of the immune system, on the blood-brain barrier (BBB), microglia, astrocytes, oligodendrocytes and neurons, potentially open the way for a plethora of therapeutic actions on different targets that could aid the management of MS. As such, cannabinoids could have an important impact on the outcome of MS in terms of the resolution of inflammation or the potentiation of endogenous repair in the central nervous system (CNS), as witnessed in the EAE, TMEV-IDD and toxic demyelination models, and through other *in vitro* approaches. In this mini review article, we summarize what is currently known about the peripheral and central effects of cannabinoids in relation to the neuroinflammation coupled to MS. We pay special attention to their effects on remyelination and axon preservation within the CNS, considering the major questions raised in the field and future research directions.

## Introduction

In the traditional pharmacopeia of human history, both recreational and medicinal uses of the Indian hemp *Cannabis sativa L*. have been described for several centuries. Introduced into Western medicine by William O’Shaughnessy in 1838 to treat a variety of conditions, including rheumatic pain and epilepsy (Russo, 2017), the use of cannabinoids (CBs) in clinical practice entered a period of latency and oblivion due to political barriers and problems in establishing quality control. Nevertheless, this did not stop researchers from demonstrating the important benefits that they may potentially be gained from their therapeutic administration. Starting with the isolation of the first cannabis compound in 1899 (Cannabinol; Dunstan, 1989), more than 120 phytocannabinoids (pCBs) have since been isolated, including the most abundant tetrahydrocannabinol (THC) and cannabidiol (CBD; Morales et al., 2017). A significant revolution began in the early 1990’s with the discovery of endogenous cannabinoid receptors (CBRs), CB1R (Matsuda et al., 1990) and CB2R (Munro et al., 1993), along with the finding of the two major endogenous lipid mediators referred to as endocannabinoids (eCBs): anandamide (AEA; Devane et al., 1992) and 2-arachidonoylglycerol (2-AG; Mechoulam et al., 1995; Sugiura et al., 1995). The activity of eCBs depends on the activation of classical and non-classical CBRs, and on a sophisticated regulatory system mediated by biosynthetic and hydrolytic enzymes involved in the generation and degradation of eCBs (Morales et al., 2017). In addition, distinct transporters mediate the movement of eCBs, both intracellularly and across the plasma membrane, further controlling the availability of eCBs in the cellular milieu (Maccarrone et al., 2014). THC, 2-AG and AEA share homologies in their three-dimensional structure, despite displaying certain chemical differences (Maccarrone et al., 2017), a resemblance that explains why pCBs bind to the same cellular receptors that recognize eCBs (Friedman et al., 2019).

## Physiological Context of Cannabis-Based Therapy: The Endocannabinoid Signaling System

The effects of CB-based medicines depend on a refined eCB signaling system comprised of the membrane and intracellular receptors that ultimately determine the cell fate and survival outcomes in multiple sclerosis (MS), and disease progression. CB1R and CB2R are coupled to G proteins, and they trigger multiple signal transduction pathways that can lead to the inhibition of cAMP formation, as well as the modulation of ion channels, nitric oxide synthase, extracellular regulated kinases or β-arrestin (Maccarrone et al., 2017). The CB1R is the most abundant G protein-coupled receptor (GPCR) in the human brain. CB1R is also present in the spinal cord and peripheral nervous system (PNS), predominantly located at neuronal synapses where it is responsible for suppressing synaptic transmission through eCB-mediated retrograde signaling (Araque et al., 2017). As eCBs can move easily across cell membranes and reach intracellular compartments, it is especially relevant that CB1Rs have been seen to be functional in neuronal mitochondrial membranes modulating bioenergetics processes (Bénard et al., 2012). CB1Rs can also be found in astrocytes, oligodendrocytes and microglia, albeit less prevalent (Zou and Kumar, 2018), as well as in other body tissues outside the central nervous system (CNS), like the gastrointestinal (GI) tract, liver, heart, adipose tissue, bone, skin, eye, skeletal muscle and the reproductive system (Maccarrone et al., 2015). CB2R expression has been described in the PNS, GI tract, cardiovascular system, liver, bone, adipose tissue and reproductive system, yet it has classically been most closely associated with cells of the immune system, including microglia (Maresz et al., 2005; Miller and Stella, 2008; Cabral et al., 2015). A variety of studies have shown that CB2Rs are also present in the brain, although much less prominent than CB1Rs. CB2R mRNA is expressed by neurons in specific brain areas, including the hippocampus, cerebral cortex, cerebellum, globus pallidus, Nucleo accumbens and dorsal striatum (Lanciego et al., 2011; Zhang et al., 2014; Stempel et al., 2016). CNS CB2Rs are highly inducible and they are mainly associated with the anti-inflammatory and immunomodulatory activity of CBs (Miller and Stella, 2008; Correa et al., 2010). Non-CBRs, like the orphan GPCR GPR55 (Moriconi et al., 2010), the transient receptor potential vanilloid 1 (TRPV1; Xia et al., 2011), and the peroxisome proliferator-activated receptor (PPAR) α and γ (Pistis and Melis, 2010), may also mediate the activity of pCBs and eCBs in the body.

## THC/CBD (1:1) in the Management of Spasticity and Pain Associated With MS

Over the past two decades, much interest has been generated in the therapeutic potential of CBs for the management of neurological disorders and pain. It is worth noting that the use of cannabis in clinical practice required preclinical studies to determine the preliminary safety, pharmacokinetics, toxicology, and efficacy of CBs. As such, the safety, efficacy and consistency of cannabis-based medicines have been clearly demonstrated, leading to regulatory approval for their use to manage spasticity in MS and in Dravet’s and Lennox-Gastaut’s syndromes (Russo, 2018). The first study of CBs in MS was published in 1981 on the basis of the sporadic improvement claimed by spastic patients after cannabis inhalation and the inhibition of polysynaptic reflexes by THC in animal studies (Petro and Ellenberger, 1981). Thereafter, other clinical studies were performed to assess the effects of CBs in the relief of MS symptoms, using different plant-derived or synthetic CBs, and different routes of administration. However, it was difficult to infer the potential beneficial effects of cannabinoids in these initial reports (Rog, 2010). Since 2002, many randomized, controlled clinical trials of cannabis-based medicines have been completed, with the Cannabis in MS (CAMS) trial the largest to date, providing limited evidence of the effects of CBs in spasticity (Zajicek et al., 2005). The CUPID study in primary and secondary MS showed that Dronabinol has no effect on the progression of the disease even in a long term followed study (Zajicek et al., 2013). Although the FDA has approved Epidiolex^®^ (oral formulation of 99% pure plant-derived CBD) for the treatment of different forms of epilepsies and CBD has beneficial effects in animal models of MS (Kozela et al., 2011; Mecha et al., 2013), no clinical trials have been proposed for this disease.

Preliminary studies with different plant-derived CB preparations, including THC, CBD or both THC and CBD in a 1:1 ratio, showed improved pain relief, bladder control, muscle spasms and spasticity in the MS patients administered THC and CBD (Wade et al., 2003). This prompted trials to be carried out with the THC/CBD mixture, which led to the approval of cannabis-based medicine for the management of spasticity, neuropathic pain and bladder dysfunction associated with MS in 2011 (Maccarrone et al., 2017). Sativex^®^ (GW Pharmaceuticals Limited, Salisbury, UK) is a 1:1 mixture of THC and CBD (27 mg/ml THC and 25 mg/ml CBD) that contains less than 10% of other cannabis compounds, terpenes and flavonoids that may be present in the plant, and that might influence the actions of the main cannabinoids (Russo, 2011). This mixture is formulated and delivered as an Oromucosal spray to achieve rapid absorption into the systemic circulation, by-passing metabolism through the liver (Rog, 2010). Studies for the management of resistant MS spasticity have established that the first 6 weeks are enough time for identifying those patients in which Sativex^®^ can be effective (Messina et al., 2017).

## The First Line of Evidence: Alterations to the Endocannabinoid System in MS and Animal Models

The first evidence for the potential benefits of cannabis-based medicines in the management of MS came from the alteration to different components of the eCB system in patients and animal models of MS. Although these results are often controversial as they might depend on disease activity and methodological variables, the increase in eCBs can potentially limit the inflammatory processes ongoing in the CNS. As such, more AEA was found in the CSF of relapsing MS patients (Di Filippo et al., 2008), as well as in the plasma (Jean-Gilles et al., 2009) and peripheral lymphocytes, and there was an association between increased synthesis and reduced degradation of eCBs in MS (Centonze et al., 2007). A reduction in the expression of one of the enzymes responsible for eCB degradation, FAAH (fatty acid amide hydrolase), was detected in the blood of secondary progressive MS patients (Jean-Gilles et al., 2009). Interestingly, an induction in the expression of CB1R, CB2R, and FAAH was described in glial cells within demyelinated plaques of MS patients, supporting a role of the eCB system in the disease pathogenesis (Benito et al., 2007). There is more AEA in inflammatory lesions of patients with active MS (Eljaschewitsch et al., 2006) and in the brains of mice with Experimental Autoimmune Encephalomyelitis (EAE; Centonze et al., 2007), whereas less eCB was detected in different brain areas when EAE was induced in rats (Cabranes et al., 2005). In terms of CBRs, less CB1R was found in the brain of EAE rats (Cabranes et al., 2005), while more CB2R was evident in the spinal cord of TMEV-IDD mice (Loría et al., 2008).

## Second Line of Evidence: Immunomodulatory Actions of Cannabinoids and Their Effects on the Blood-Brain Barrier

MS is a multifactorial disease and it is widely accepted that CNS neuroinflammation is responsible for demyelination. As such, the main objective in patients management is the modulation of different components of the immune system through first and second-line therapies that include Interferon β (IFNβ), Glatiramer Acetate, Fingolimod, and Dimethyl Fumarate. These and other treatments involve the blockade of lymphocyte homing to the CNS (e.g., natalizumab, rituximab, ocrelizumab and alemtuzumab), a reduction in the B lymphocyte counts through the use of humanized antibodies, the dampening of lymphocyte proliferation (e.g., Teriflunomide), and the promotion of an anti-inflammatory profile of immune cells (Yanagawa et al., 1998; Ziemssen and Schrempf, 2007; Reder and Feng, 2014).

Despite controversial results, there is strong evidence for a therapeutic effect of CBs in animal models of MS, given that the exogenous administration of pCBs, eCBs and synthetic CBs ameliorates motor symptoms and improves the disease outcome by decreasing neuroinflammation; reviewed in (Chiurchiù et al., 2018). Cannabis-based therapies are thought to dampen the immune responses associated with a plethora of neuropathological conditions by selectively targeting CB2Rs expressed by immune cells, including that of CNS resident microglia. Evidence is accumulating that CBs modulate immune responses during inflammatory processes and their effects have been studied in many disease models of MS. Animal studies show that CBs exert their immunomodulatory properties by targeting various cell types: (i) inducing apoptosis in peripheral and central T cells (Palazuelos et al., 2008; Sánchez and García-Merino, 2012); (ii) promoting a reparative activation state of microglia and macrophages (Mecha et al., 2016, 2018); (iii) inhibiting the expression of adhesion molecules by cerebral endothelial cells (Ni et al., 2004; Mestre et al., 2009); (iv) suppressing T cell proliferation (Lombard et al., 2007; Rieder et al., 2010); and (v) inhibiting pro-inflammatory cytokine/chemokine production while increasing anti-inflammatory cytokines (Kozela et al., 2011).

The BBB shields the CNS from toxins and immune cells in the blood, and it allows molecules, ions, and cells from the brain to be passed into the blood, ensuring an adequate milieu is maintained for neuronal and glial cell functions. Immune surveillance takes place in physiological conditions as a necessary aspect of neuroimmunity (Ousman and Kubes, 2012) and new evidence suggests the existence of a meningeal lymphatic system (Da Mesquita et al., 2018). As CNS inflammation occurs in the early stages of MS, it boosts the recruitment of activated immune cells by promoting adhesion and transmigration across the activated BBB (Ransohoff, 1999), a multi-step process that requires the induction of adhesion molecules (ICAM-1, PECAM-1), chemokines (CCL2) and integrins (α4 integrin, β1 integrin: reviewed by Engelhardt et al., 2017). In homeostatic conditions, the CB2R is expressed at low levels in endothelial cells of the BBB (Schley et al., 2009), as well as *in vitro* (Mestre et al., 2006). During neuroinflammation, the brain endothelium enhances the expression of CB2R, a mechanism perhaps designed to regulate endothelial activation since these cells produce further eCBs upon inflammation (Golech et al., 2004; Ramirez et al., 2012). In the TMEV-IDD model of MS, administration of the non-selective CB1R/CB2R agonist WIN55, 212-2 inhibits the infiltration of leukocytes into the CNS (Arévalo-Martín et al., 2003; Ni et al., 2004) and ameliorates disease progression (Croxford and Miller, 2003). Furthermore, this agonist suppresses ICAM-1 and VCAM-1 expression in the brain endothelium, which is concomitant with reduced CD4^+^ T lymphocyte infiltration into the CNS and the ensuing neuroinflammation (Mestre et al., 2009). Indeed, CB2R agonists dampen the induction of ICAM-1 and VCAM-1 in brain endothelial cultures exposed to proinflammatory mediators (Ramirez et al., 2012). A role for CB1R in endothelial cells has also been described, as AEA administration inhibits the induction of VCAM-1 in endothelial cells after TMEV infection and decreases leukocyte transmigration in an *in vitro* model of the BBB, an effect that is absent in the presence of CB1R selective antagonists (Mestre et al., 2011). Finally, other mechanisms independent of CB signaling have been proposed, since effects on the BBB and the infiltration of leukocytes into the CNS are also observed when the pCB, CBD is administered *in vivo* or *in vitro* (Mecha et al., 2013; Hind et al., 2016).

## Third Line of Evidence: CNS Repair Mechanisms Mediated by Cannabinoids

Substantial advances have been made in the past decades to control the exacerbated immune activity associated with MS, yet current treatments have yet to halt the progression of the disease or to enhance endogenous repair mechanisms in the CNS. Neuroprotective therapies, and those targeting oligodendrocyte progenitors and other CNS cells, such as astrocytes and microglia, are likely to promote recovery and prevent long-term neurodegeneration. Indeed, the neuroprotective effects of CBs have been confirmed in different models of injury and CNS disease, like Alzheimer’s Disease (Martín-Moreno et al., 2012; Schubert et al., 2019), stroke (Zarruk et al., 2012; Kolb et al., 2019), ischemic injury (Fernández-López et al., 2007), Parkinson’s Disease (García et al., 2011) and ALS (Rodríguez-Cueto et al., 2018). In the TMEV-IDD model of progressive MS, the administration of synthetic CBs (Arévalo-Martín et al., 2003) or pCBs (Mecha et al., 2013; Feliú et al., 2015) has been associated with an improvement in neurological defects, also observed by inhibiting selective AEA uptake (Ortega-Gutiérrez et al., 2005) or the enzymatic hydrolysis of 2-AG (Feliú et al., 2017). In this latter study, both remyelination and axon preservation was showed, while chondroitin sulfate proteoglycans diminished through the involvement of CB1R and CB2R. In addition, 2-AG administration or inhibition of its hydrolysis favors oligodendrocyte precursor cell (OPC) differentiation (Gomez et al., 2015) and, by diminishing the excitotoxicity of oligodendrocytes, demyelination is prevented in the EAE (Bernal-Chico et al., 2015) and the cuprizone model (Manterola et al., 2018).

## Conclusions and Future Directions

**Figure 1:**
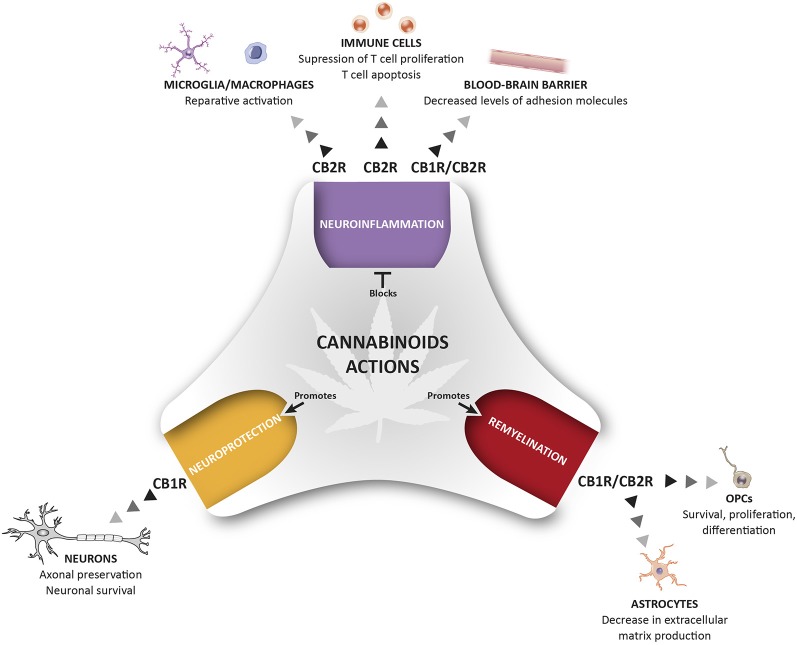
Therapeutic potential of cannabinoids (CBs) for the management of pathological events related to multiple sclerosis (MS). The activation of CB1R and CB2R present in different cells of the immune and nervous system control different pathological events related to MS including neuroinflammation, repair mechanisms, and neuroprotection. This holistic perspective of CBs treatment provides a multi-target medicine available for the management of MS.

The eCB system plays an important role in CNS homeostasis and neuroprotection, participating in immune control and maintaining the fine-tuned homeostatic balance of the central immune system. In MS and other neurodegenerative diseases, the neuroprotective effects of cannabinoids have been attributed to stimulation of CB1R, the most abundant GPCR in the brain, whereas CB2R, the non-psychotropic cannabinoid receptor, has almost exclusively been associated with immunomodulatory effects. This view was challenged by the discovery of functional CB2R in specific neurons and in other critical cells in MS, such as endothelial cells. Here, we have reviewed data on the pathophysiological relevance of CB1R and CB2R signaling in the context of MS (Figure 1). There is significant evidence that CB2Rs may contribute to the protective mechanisms operating at multiple levels to orchestrate homeostatic responses. However, it is extremely difficult to decipher the specific roles of CB1R and CB2R, and how they differ, particularly, in relation to the pathogenic events associated with neurodegeneration in MS. Future research will be necessary to identify the precise mechanisms triggered by cannabinoid signaling in order to regulate key homeostatic pathways in the brain. In addition, since both CB1 and CB2 may co-exist in the same cell, there is a need to define what type of interaction exists between these two receptor subtypes, and what could be its physiological and pharmacological relevance. The potential therapeutic exploitation of CB2R in MS *via* the targeting of neuroinflammation, the BBB, oligodendrogenesis, remyelination, axon preservation and neuronal survival, is likely to be of particular interest regarding neurodegenerative diseases. In summary, pharmacological activation of CB2Rs needs to be explored in-depth to develop innovative drugs that can counteract the motor and neurological deterioration in MS. The added value of this potential therapeutic strategy appears to be the reduced risk of psychoactive effects associated with CB2R manipulation.

## Author Contributions

MM and CG designed and wrote the manuscript. FC-S illustrated Figure 1. LM and AF revised the manuscript.

## Conflict of Interest

The authors declare that the research was conducted in the absence of any commercial or financial relationships that could be construed as a potential conflict of interest.
